# Ethnic boxes: the unintended consequences of Habsburg bureaucratic classification

**DOI:** 10.1080/00905992.2018.1448374

**Published:** 2018-06-01

**Authors:** Rok Stergar, Tamara Scheer

**Affiliations:** a Department of History, Faculty of Arts, University of Ljubljana, Ljubljana, Slovenia; b Ludwig Boltzmann Institute for Historical Social Science, University of Vienna, Vienna, Austria; c Institute for East European History, University of Vienna, Vienna, Austria

**Keywords:** nationalism, Habsburg Empire, bureaucracy, classification, Central Europe

## Abstract

The classificatory efforts that accompanied the modernization of the Habsburg state inadvertently helped establish, promote, and perpetuate national categories of identification, often contrary to the intentions of the Habsburg bureaucracy. The state did not create nations, but its classification of languages made available some ethnolinguistic identity categories that nationalists used to make political claims. The institutionalization of these categories also made them more relevant, especially as nationalist movements simultaneously worked toward the same goal. Yet identification with a nation did not follow an algorithmic logic, in the beginning of the twentieth century, sometimes earlier, various nationalisms could undoubtedly mobilize large numbers of people in Austria–Hungary, but people still had agency and nation-ness remained contingent and situational.

## Introduction

In the last decade, scholars have argued against an ethnicist interpretation of the formation of modern nations in the Habsburg Empire.^1^ Narratives of the unstoppable development of ethnic groups into nation-states have been questioned and rejected. Scholars have persuasively argued that nations did not evolve from ethnic communities, but were created by nationalists who first imagined them and later fashioned them into viable categories of identification.^2^ Scholars have also shown that – at least in the first half of the nineteenth century – multiple concepts of nations circulated. Alexander Maxwell, for example, argued that the establishment of the Slovak nation was never a foregone conclusion, as other equally viable ethnolinguistic concepts emerged simultaneously (Maxwell 2009). Furthermore, in their polemics against Josephinist centralism, members of provincial estates discussed nations defined by provincial borders and not by language or a putative ethnicity. Finally, Austrian patriotism offered an opportunity for the establishment of a civic Austrian national idea (Judson 2016, 63–63, 87–89). In other words, recent historiography shows that the emergence of particular nations in the Habsburg Monarchy was not an inevitable consequence of modernization or a historical necessity. On the contrary, it was a process marked by a variety of options, discontinuities, chance, and failure. Nations were established as heterogeneous populations that spoke a variety of vernaculars and lacked a common identity were transformed into imagined communities. If we slightly rephrase the title of Eugen Weber’s seminal book, peasants (and others) were slowly turned into Croats, Serbs, Czechs, Slovaks, Italians, Romanians, Poles, Ruthenians (Ukrainians), Hungarians, Slovenes, and Germans, in a process that lasted more than a century. Apparently, Joep Leerssen’s observation that “the history of nationalisms is far more complex than merely a prehistory of the contemporary states” (Leerssen 2006, 18) applies to Habsburg nationalisms as well. Therefore, we need to take a fresh look at the historical contingencies and agents involved in this process to gain a better understanding of the emergence of nations in this region.

Certainly, the main protagonists of nation-building were nationalists. They imagined “their” nations; wrote national pamphlets, programs, and declarations; drew borders on maps; established nationalist movements with their myriad associations; and made every effort to nationalize the inhabitants of the Habsburg Empire – however slowly and often not entirely successfully. Quite rightly, the nationalists’ role in constructing nations and the nationalization of the masses is the focus of older and more recent scholarship.^3^ But other important actors in nation-building have also been discussed, including the Habsburg state. Traditionally, historians have claimed that the Habsburg state was antagonistic toward nationalist movements. Yet some historians have already pointed out that the empire was both an antagonist and an enabler of nationalism. Gary B. Cohen argued almost a decade ago, “it was the Habsburg state that made possible the growth of political space and institutional venues for the development of a modern civil society and, with that, nationalist politics” (Cohen 2007, 245, 274).

Our argument goes a step further. We argue that the classification efforts that accompanied the modernization of the Habsburg state were in fact key to establishing, promoting, and perpetuating national categories of identification. To some extent, the Habsburg Monarchy even functioned as a (multi)nationalizing state in the last years before World War I.^4^ Certainly, this was inadvertent, and often contrary to the intentions of the Habsburg bureaucracy. In his very informative analysis of institutionalized ethnicity, Siniša Malešević has drawn attention to the “important distinction … between the normative or official ideological narrative of the particular political order and its operative, which is to say institutionalized, counterpart” (Malešević 2006, 161). In the Habsburg Empire, the supranational *viribus-unitis*-empire was the official narrative but the classification and categorization efforts of bureaucrats were its operational counterparts. As arbitrary as the processes might have been, classification and categorization helped pigeonhole the inhabitants of the Habsburg Empire into neat national categories. This had real consequences, not least because, as Robert Darnton reminded us, “pigeon-holing is … an exercise in power” (Darnton 1984, 192, 193; also Foucault 1970). In short, the boxes used in bureaucratic forms and questionnaires by the modernizing state throughout the nineteenth century turned into ethnic boxes. With the frequent use of national and quasi-national categories in various classificatory endeavors, the Habsburg state helped propagate the notion that its inhabitants belonged to one or another nation that could be defined by language use.

The process of nationalization through Habsburg bureaucratic classification was slow but ongoing. During the first stage, the state’s pragmatic effort to standardize languages to simplify the work of the bureaucracy was decisive. It was of great importance that some vernaculars were not standardized and the use of some existing written languages was discontinued. Therefore, some languages were reduced to the status of a dialect and could – according to the prevailing perception of the period – no longer serve as a basis for the establishment of a nation. In the second phase, an increasing number of bureaucratic structures categorized the population for several reasons. Repeatedly being classified by bureaucrats familiarized people with the newly established linguistic and/or national categories. Finally, in the third phase, these national categories were embedded in the legal framework of some imperial and provincial institutions. Consequently, nation-ness was not a matter of choice anymore; it was a legal requirement. Citizens were sometimes even assigned a nationality, influencing their access to schools, voting rights, and many other aspects of everyday life.

## The standardization and classification of languages and their impact on the early stages of nation-building

As was noted above, Joep Leerssen argues that the prehistory of nationalisms was complex and that there were alternatives to the nations that eventually emerged (Leerssen 2006, 18). Indeed, in the last decades of the eighteenth and the first decades of the nineteenth century, several alternative concepts of nations started to take shape in the Habsburg Monarchy. Some were based on the widespread belief in the existence of ethnic groups that only needed to be awoken and then transformed into modern political communities. Other concepts, as we have mentioned, envisioned the transformation of all the inhabitants of a province or the entire empire into a nation. Many of these alternatives gained scant support and fizzled quickly. National movements, based on different concepts, emerged in only a few cases. To use Miroslav Hroch’s terminology: many possible and envisioned nations never made it from Phase A, the so-called period of scholarly interest, to Phase B (Hroch 1985).^5^ For example, when Emperor Leopold II reconvened the Provincial Diets following the death of his brother and predecessor, Joseph II, the Carniolan Estates claimed to be the representatives of a Carniolan nation in their 1790 *gravamina* [List of Complaints]. They demanded that all provincial officials be Carniolans because locals used their own language and were of a different origin (Zwitter 1990, 100, 101). A few decades earlier, Marcus (Marko) Pohlin, a patriotic Catholic monk, had tried to persuade his compatriots that their Carniolan language was as useful as any other and that they should be proud of it (Pohlin 1768). Despite these efforts, a Carniolan nationalist movement never emerged and no Carniolan nation exists today. Instead, a few decades later the Slovene nationalist movement established itself in Carniola and some neighboring provinces.

Until recently, scholars explained such developments within an ethnicist framework; the success of a national movement was supposedly a necessary consequence of its reliance on a pre-existing ethnic group. Failure to create a new nation was therefore the unavoidable result of a group lacking a shared ethnic foundation. However, as we mentioned in the introduction, recent research has persuasively demonstrated that the nations in Central and Southeastern Europe were not a continuation of earlier ethnic communities (King 2001; Norton 2007; Maxwell 2009; Pantelić 2011; Malešević 2017). Slovene nationalism did not prevail because it was a continuation of a hypothetical ethnic community. In fact, a Slovene ethnic community never existed. The idea of a Slovene nation emerged in the first decades of the nineteenth century based on a novel categorization of languages (Hösler 2006; Kosi 2013; Kosi and Stergar 2016).

If the existence of an ethnic core does not explain the success of some and the failure of other nationalisms, it is necessary to look for other decisive factors. We argue that in the late eighteenth and early nineteenth century, during the early stages of nation-building, the classification of languages became crucial to the formation of nations. When the understanding of nationality as an attribute that could be objectively determined by language use became commonplace, distinguishing between language and dialect became much more than just a scholarly exercise. On the contrary, it imposed arbitrary hierarchies and deeply influenced the understanding of what can become a nation and what cannot (Kamusella 2009, 23–51 and passim; Maxwell 2009, 142; Kamusella 2016).

Even though linguists and other scholars played a crucial role in classifying and categorizing languages, the role of the state should not be overlooked. Josef Dobrovský, August Ludwig von Schlözer, Bartholomäus Kopitar, and Pavel Jozef Šafarik were among the scholars classifying vernaculars as dialects or languages. These linguists’ arguments were often arbitrary, but they were bolstered by the impression that they were propagating the unbiased results of scientific inquiry. The impact of scholarly classifications would have been far smaller if they were not sanctioned by Habsburg bureaucrats. Again, language classification was performed to fulfill the monarchy’s pragmatic needs. When Joseph II made German the official language to streamline the administration of his empire, state authorities grew increasingly aware of the importance of local vernaculars for their educational efforts and communication with their subjects. Therefore, they pragmatically supported the standardization of non-German literary languages (Vocelka 2001, 241, 242, 386).

One outcome of standardization was a significant reduction in the number of officially used written languages at the end of the eighteenth and in the first half of the nineteenth century. At that time, the Habsburg state put its weight behind efforts to develop standard languages that could be used across provincial borders or it selected one of the existing standards over another. This move was purely pragmatic and was certainly not supposed to cultivate nations within the empire. After all, this happened at a time when there were no policies to deal with emergent nationalisms; in fact, most Habsburg statesmen and top bureaucrats underestimated their importance (Clewing 2001, 170–173). Imperial bureaucrats were simply eager to reduce the number of languages in their territories to simplify their work. However, as an entirely unintended consequence, the number of languages that could serve as the basis for the conceptualization of nations was reduced. Therefore, pragmatic policy heavily influenced the earliest phase of nation-building.

To demonstrate how this worked, we will examine the case of the Slovene language. In the beginning of the nineteenth century, the idea of a single Slovene language emerged. The linguist Bartholomäus (Jernej) Kopitar claimed that the Slavic vernaculars of Carniola, the Austrian Littoral, southern Carinthia, and Lower Styria were just dialects of a single South Slavic language. Previous authors had mostly distinguished three distinct Slovene languages, Carniolan, Styrian, and Carinthian (Kosi 2013, 151–167). Kopitar’s interpretation quickly became popular among intellectuals in all those provinces and they made every effort to amalgamate existing provincial literary languages into one (Orožen 1996; Hösler 2006, 152–188; Kamusella 2009, 294, 295; Kosi 2013, 170–189). The new standard language rather quickly established itself in literature and, perhaps most importantly, in the new weekly *Kmetijske in rokodelske novice* [Agricultural and Artisanal News] published from 1843 on. That created an Andersonian unified field of exchange and communication. Since the notion that a separate language denotes a separate nation had also been spreading, the idea of a distinct Slovene nation began to take shape alongside the Slovene language’s emergence (Hösler 2006, 244–270; Kosi 2013, 236–360).

However, not all local intellectuals were willing to abandon their provincial standard languages. The majority in South Styria continued to use and develop their version of Slovene into the first half of the nineteenth century. At first, Styrian authorities supported these developments, but at Vienna’s suggestion the provincial government in Graz forbade the use of the Styrian Slovene orthography and language in schools in 1838. So the use of this written language ended (Jesenšek 2015, 108, 214, 215, and passim).^6^ Although the intervention of Habsburg authorities in favor of the new unified Slovene standard language was certainly motivated by a wish to simplify the linguistic situation, it had political implications. It opened the door for the assertion of Kopitar’s unified Slovene language and, consequently, Slovene nationalism in South Styria. It also showed that the Habsburg state increasingly saw the Slav speakers of the Austrian Littoral, Styria, Carinthia, and Carniola as a distinct community. When non-German editions of the *Reichsgesetzblatt* (The Official Law Gazette) were implemented in 1849, this classification was already well-established; a single Slovene edition for all the Slav speakers in the above-mentioned provinces was published.

The case of the Slovene language was not exceptional. During the first half of the nineteenth century, the Habsburg state was often involved in the standardization of languages that later provided a basis for the national classification of its inhabitants. To be clear, most of the work was done by linguists, but the state acted as an arbiter. It allowed the use of certain standards in schools and used them in its communication with the public. In Dalmatia, for example, the state started abandoning the use of the local Ikavian version of Illyrian (i.e. Serbo-Croat) and began employing the version used in Croatia and Slavonia (Clewing 2001, 355). In 1849, the process of standardization culminated in the publication of nine versions of the *Reichsgesetzblatt*: German, Italian, Hungarian, Czech, Polish, Ruthenian (Ukrainian), Slovene, Serbo-Croat (in a Latin and a Cyrillic edition), and Romanian (*Allgemeines Reichs-Gesetz- und Regierungsblatt for das Kaiserthum Österreich* 1849, VI). At that point, the Habsburg language classification was mostly finished; at the imperial level, only Slovak was later added as an independent language. Other languages – such as Friulian, Hebrew, or Yiddish – were never officially recognized as such.

The classification was largely arbitrary and was certainly not a reflection of the contemporary situation. For example: the *Reichsgesetzblatt* had a single Czech edition – called “Bohemian (at the same time Moravian and Slovak)” – and no separate Slavic Moravian edition, although Moravian had a distinct literary tradition (Kamusella 2009, 105, 488, 489). There was also no separate Slovak edition even though several Slovak scripts were in use at that time (Maxwell 2009, 117–165 and passim). Not only were some written languages ignored but the classification scheme used to define languages was not unanimously accepted among scholars. There were many alternative classifications of Slavic languages, for example. Some linguists classified Slovak as a language, others as a dialect of Czech, Czechoslovak, or even Polish. Some considered Slovene a language, while others were convinced that it was only a dialect of the Illyrian (i.e. South Slav) language (Maxwell 2006, 142, 2009, 103). On top of that, many contemporaries were convinced that all the Slav vernaculars were dialects of a single Slav language (Maxwell 2009, 80–100).

However, the state approved classification of languages had a significant impact. Nine linguistic categories from the 1849 *Reichsgesetzblatt* were used in bureaucratic procedures and informed political discussions. They provided a useful framework for the nationalists and their attempts to nationalize previously non-national inhabitants of the Habsburg Empire. The Habsburg state’s engagement in the standardization and classification of languages during the first half of the nineteenth century laid a foundation for the establishment of ethnic boxes in the following decades.

## Classification as a vehicle of nationalization

In 1849, the same year the Habsburg state sanctioned the linguistic classification it had helped to shape in the previous decades, legislators prepared the draft of the Austrian constitution that included a paragraph on the equality of the Habsburg *Volksstämme* [nationalities] and their right to cultivate their languages in public. This constitution was never promulgated but the paragraph found its way into the Austrian December Constitution of 1867 (Stourzh 1985, 17–83). In both cases the nationalities were not defined or named, but most parliamentarians assumed they could be defined by language use. They followed the prevalent equation of language groups with nations, a concept that had a long tradition and gained popularity from the turn of the nineteenth century forward. Many key authors of the Enlightenment had already claimed that a person’s spoken language denoted his/her nationality, but the idea became especially influential throughout Central Europe after Johann Gottlieb Fichte popularized it in his *Reden an die deutsche Nation* (Addresses to the German Nation) at the beginning of the nineteenth century (Kostantaras 2016). In 1848 and 1849, some nationalists were still advocating for nationality based on provincial identity or the establishment of an Austrian nation. These voices were in the minority and mostly fell silent in the following decades (Judson 2016, 204, 205). Similarly, the idea of a multilingual Hungarian nation was mostly superseded by the idea of Hungarians who spoke Magyar exclusively (Gal 2011, 36–41).

The arbitrary state classification of languages had important political consequences; regardless of its pragmatic, bureaucratic motives, the state implicitly recognized and legitimized some nationalisms and made others almost impossible to promote. One consequence was, as Elke-Nicole Kappus noted in her analysis of Habsburg policies in Istria, the institutionalization of “categories in terms of which the people would increasingly model their sense of belonging, loyalty, and identity well before the arrival of the ‘nation state’” (Kappus 2012, 324). The state’s inadvertent participation in the establishment of national categories was just one part of the equation. Even more important, it put the categories into active use to label and manage the empire’s population. Bureaucratic forms that required people to label themselves using one of the prescribed categories were used increasingly during the period. There was no option to describe oneself as a non-national or as an “amphibian” in the forms; there were no Roma, no Moravians or Hanáks, nor Friulians or Dalmatians, no Bosniaks, and – let us not forget – no Austrians either.

The decennial censuses help illustrate how forms and their categories influenced the process of nationalization in the Habsburg monarchy, both in Austria and in Hungary. From 1880 on, the inhabitants of the Austrian half of the Dual Monarchy had to declare their *Umgangssprache* [language of daily use] in the census forms from among the following: “German, Bohemian-Moravian-Slovak, Polish, Ruthenian, Slovene, Serbian-Croat, Italian-Ladino, Romanian, and Hungarian.” These categories were based on the classification established in 1849 and each respondent could choose only one (Reichsgesetzblatt, 6 August 1880, 372).^7^ Here, as in many other respects, the census did not reflect linguistic reality. On the contrary, the use of many vernaculars was reduced to a set of predetermined linguistic categories; bilingualism was eliminated (King 2002, 58, 59). However, the census did not simply reduce diversity, it also amplified it (Göderle 2014). Namely, these reductionist answers were often presented and understood as a description of the population’s national distribution. Many people equated linguistic categories with national ones and interpreted the results of the census accordingly (Brix 1982). It was by no coincidence that Jewish nationalists worked hard to make the Austrian state recognize Yiddish as one of the approved categories in the census; without it the claim to Jewish nationhood could scarcely be made (Shanes 2012, 115). Because language use was understood as a reflection of nationality, the results of the census buttressed the illusion that the entire population of the Habsburg Monarchy belonged to one of the nine ethnolinguistic nations. Nationality was, in the words of Wolfgang Göderle, one of the “categories that applied only to a part of the population [but] had to be extended to the entire population” (King 2002, 59; Göderle 2016, 81).

The state statisticians insisted that these results should only be understood as intended: as a snapshot of language use. However, they were also well aware of the fact that the data they presented were used by the public and scholars as a proxy for nonexistent nationality statistics (Bureau der k.k. Statistischen Zentralkommission 1912, 58–59). Besides, the state itself was not entirely consistent in its bureaucratic practices. The imperial army, for example, published its own annual statistics. The army basically adopted the taxonomy used in the civil census, although it counted Czechs and Slovaks separately. Contrary to the civil census where people were asked for their language of daily use, the military asked soldiers about all their language skills. The results were presented as the national composition of the rank and file in the *Militärstatistisches Jahrbuch* (Military Statistical Yearbook) (published 1870–1911), which categorized soldiers, non-commissioned officers, and officers, in 10 *Nationalitäten* (nationalities). Paradoxically, the self-styled last bastion of the unitary empire thus promoted the impression that all the inhabitants of Austria–Hungary had distinct national identities (Stergar 2012, 292, 293; Scheer 2016, 66–67).

There was a third major effort where citizens were counted: the Hungarian census. The first census conducted in 1867 did not have any data on language or nationality, but the Hungarian statistical bureau started to report the “mother tongue” of the inhabitants from the second census in 1880. However, since Hungarian politicians feared that statistics based on the residents’ mother tongue would show the numerical weakness of Magyar speakers within the realm of St. Stephen, the census included a question on other spoken languages (Gal 2011, 42; Varga 2014, 967). Therefore, its results reflected the linguistic diversity slightly more than the Austrian census. Respondents were still limited to a pre-set taxonomy of languages. The categories used in the Hungarian census were similar to the Austrian, but there were some important differences. Slovak was recognized as a separate language in Hungary, and Serb and Croat were sometimes separated too (Varga 2014, 968). For several years, certain local glossonyms were allowed on the census forms: for example, Bunjevci and Šokci, (South-Slav-speaking inhabitants of East Slavonia and Southeastern Hungary) could declare that they spoke a separate language from other Slavs (Kamusella 2009, 239). Despite these differences, one aspect of the results was identical to the Austrian census. The entire population was divided into categories that were widely understood as national at the time and the impression was created that everyone in the empire belonged to a nation.

The censuses did not simply misrepresent social reality, above all they shaped it. First, completing census forms familiarized the population with national categories of identification that were still a novelty for many. After all, at that time “the very concept of ‘nation’ meant nothing” to large parts of the population (Judson 2006, 5). An analysis of the 1880 census forms for Ljubljana/Laibach clearly shows that several respondents were not familiar with the classification choices they were supposed to use and used older folk taxonomies instead. Some described their language of everyday use as Carniolan or just Slav, a man distinguished between his Slovene and his wife’s *Windisch* (the traditional German term for non-Carniolan Slovene), and some respondents claimed two languages of daily use (Valenčič 1974, 300–303). Despite the nationalists’ pressure and the official instructions, a small minority of respondents in Prague also reported two languages (Cohen 2006, 67). Similarly, in Hungary, respondents often described their language as “Saxon, Swabian, *Jargon* (Yiddish), *Landler* or simply *unsere Sprache* (our language)” instead of the officially allowed German (Berecz 2013, 25).

Second, the census was an opportunity to choose. With the strengthening of nationalist movements, it increasingly became an opportunity to affirm loyalties (Cohen 2007, 261). Not by coincidence, nationalists only increased their activities at the time of the census, which they saw as an opportunity to promote nationhood (Judson 2006, 15). In short, as in other so-called multinational states, the census helped affirm categories of identification even though it was supposedly only measuring population statistics (Kertzer and Arel 2002; Anderson 2006, 168–174; Yosmaoğlu 2006). It was one of the events that invoked and evoked nations and thus helped the efforts of nationalists to reify them (Brubaker 2006, 10).

Being decennial, however, the census’ impact was limited. Other classificatory efforts were more frequent or even permanent and therefore more central. The use of classification in schools was extremely important. First, it forced parents and/or schoolchildren to choose among increasingly limited options. The example of a high school (*Gymnasium*) in Marburg/Maribor illustrates the point well. Until 1860, schoolchildren were classified in three categories: German, Slovene, and bilingual (*Utraquisten*) or Slovene-German. After that year, only two options (German or Slovene) remained even though many pupils were bilingual and did not feel comfortable with a binary choice (Almasy 2014a, 160–162, 2014b, 142). Even more importantly, this classification had practical consequences. All over the empire, pupils in different categories were not only taught by different teachers, often nationalists, but the curricula they were taught differed greatly too. On the one hand, pupils were subjected to patriotic propaganda focused on the ruling dynasty. But on the other hand, Czech-speaking children were not only told they were Czechs, but they also read other books and learned about other historical figures than, say, German-speaking children. The curricula were largely nationalized (Bruckmüller 1999, 2003, 2007).

These practices helped construct ethnic boxes by reinforcing the boundaries between groups. Autobiographical sources clearly show the consequences of classification in schools. Josip Sernec, for example, was raised in a predominantly German-speaking bilingual family in Lower Styria and later became one of the most prominent Slovene nationalist politicians in the province. Sernec acknowledged in his memoirs that he only “became aware of his Slovene nationality” in middle school and that this largely happened under the influence of his teachers. He likely would not have developed a Slovene identity if he had not been categorized as a Slovene and put into a Slovene class (Sernec 1927, 2–8).

Classification was consequential for other people living in bilingual areas as well. The linguistic classification of vernaculars as dialects of a language promoted the notion that their speakers were members of particular nations. Teaching children the standard language in schools made communication among supposed co-nationals easier – or even possible. Before learning High German became widespread in Transylvanian Saxon schools, speakers who used different Saxon vernaculars often had to resort to using Romanian to talk to one another. In response to these linguistic challenges, the teaching of German was promoted to make Saxons a part of the German nation (Berecz 2013, 87, 88). Saxon schools were private and the classification of Saxons as Germans was a result not of state policies but of the Lutheran Church, yet it paralleled similar processes in state schools. Speakers of almost mutually incomprehensible vernaculars were told that they spoke the same language. Then the schools taught them the standard version of their language; this made the unified field of exchange and communication possible and provided the basis for the expansion of nationalism (Anderson 2006, 46). German-language schools did not necessarily turn Slovene-speaking pupils into Germans, for example, but Slovene-language schools certainly played a large role in making Slovenes out of pupils speaking different Slovene vernaculars.

Somewhat surprisingly, the Austro-Hungarian army also played an important role in the promotion of national categories based on linguistic classification. As in most other cases, this was not intentional but was a result of policies and practices that had other, more pragmatic, goals. In fact, the intentions of the army, especially after the monarchy was split in two after the Compromise of 1867 and universal military service was introduced a year later, were exactly the opposite. The army intended to serve as the “school of nation,” an institution that would instill loyalty to the emperor and the Habsburg state in recruits (Allmayer-Beck 1987, 88–96). Research has shown that such endeavors met with a degree of success (Deák 1992; Cole 2014). Even though the army was trying to make good Austrian patriots out of conscripts, it also sorted them into linguistic “ethnic boxes” to ease the challenges of training. The army invoked and affirmed national categories of identification and made them into relevant categories of everyday practice for the soldiers.

We have already mentioned “statistical nationalization” in the army and its impact. The most important mechanism in this process was the use of the so-called regimental languages, a peculiarity of the Austro-Hungarian army. As universal military service was introduced in 1868, the army formalized the use of regimental languages. German remained the sole language of command, but 10 languages (Croat or Serb, Czech, German, Hungarian, Italian, Polish, Romanian, Ruthenian/Ukrainian, Slovak, Slovene) were given a special role. When more than a fifth of recruits in a regiment spoke a common language, it became a regimental language. From that point, officers had to use that language to communicate with the recruits from that linguistic group (Allmayer-Beck 1987, 98, 99).

The aim was to make training more efficient or even possible, but there were other consequences. New recruits, speaking their native vernaculars, or often bilingual, were assigned to one of the approved linguistic categories upon entering the army. At the same time, they were also classified as members of a corresponding ethnolinguistic nation even if they did not feel they belonged to it or refused national affiliation altogether. For example, in 1902, Johann von Appel, corps commander in Sarajevo, reported to the War Ministry in Vienna that Muslim soldiers regularly complained about being categorized as either Serbs or Croats because they could not choose Bosnian as their language (Arhiv Bosne i Hercegovine, Sarajevo, Joint Ministry of Finance, Department for Bosnia and Hercegovina [ZMF], b. 16659, Appel to the War Ministry, 4 December 1902).

Such administrative categorization not only nationalized the soldiers statistically, but it could also establish or strengthen the link between language and nationality in the minds of soldiers. Moreover, serving in linguistically homogeneous units created living conditions significantly different from the usual experience of soldiers from the bilingual areas of the empire and/or bilingual families. The use of a single language – apart from some 80 German commands, of course – became the norm. The connection between language and nationality grew stronger because soldiers were regularly treated as members of a particular nation by the army. Intra- and intergroup dynamics must be considered in this case too. The formation of linguistically homogenous groups tended to fortify the dividing lines among different groups and reinforce group homogeneity. It helped spread nationalism, as an increasing number of soldiers already were nationalists when they were called up. All of this made national categories of identification more relevant, and sometimes self-evident, for the soldiers. A consequence of military linguistic policies was that being Slovene, or Czech, or German, or Slovak, became more common, and having a national identity was increasingly perceived as the “natural” order of things.

By classifying its inhabitants in ethnolinguistic categories, in schools, the army, during the census, and on other occasions, the Habsburg state helped popularize the idea that everyone could be ascribed to a single, objectively determined, and internally homogenous national group. In other words, that everyone had an ethnic box they fit in. In the process, it inadvertently helped nationalists and their countless organizations to construct and strengthen the boundaries among emerging national communities. The imperial bureaucracy joined the nationalists in the recurrent – to use Michael Billig’s phrase – “flagging” of nationhood (1995), thus helping to reproduce the nations as pertinent categories of identification.

## The Habsburg monarchy as a nationalizing state

If nothing else, the way classification influenced and promoted nationalisms was important because of its scope. The censuses, the schools, and the army enveloped a vast number of people, many more than nationalist newspapers, associations, and political parties. Nevertheless, and despite all the classification and all the efforts of nationalists, nationalisms were relevant only for parts of the population and only in some situations. Not only recent research on national indifference (Judson 2006; Bjork 2008; Zahra 2008; Struve 2014; Bjork et al. 2016) but also traditional historiography has shown that even as late as 1914 the nationalization of the masses was far from finished. For example, decades ago, the eminent Croat historian Mirjana Gross (1981) demonstrated that in Istria, Dalmatia, and Bosnia and Hercegovina, Croat nationalism began reaching the masses only after 1918, in Yugoslavia. At the beginning of the twentieth century, nationalism was still largely limited to politics and public institutions (Judson 2016, 331). Clearly, state institutions were important but their impact was limited by many other factors. For example, in his innovative book on Hungarian schools, Ágoston Berecz showed that the impact of Hungarian language education on non-Hungarian speakers was very limited; the claims of Hungarian nationalists that schools were making Hungarians out of Romanians and Saxons were overly optimistic. In places where almost everyone was speaking Romanian or Saxon vernaculars, a few years of school in Hungarian hardly made a difference (Berecz 2013, 196–219).

Above all, people still had agency and often refused to be put in boxes. Yes, they had to declare themselves monolingual on the census forms, but many still were bilingual and wanted to stay that way. In fact, many parents worked hard to make sure their children became bilingual (Judson 2006, 46–48 and passim; Zahra 2008, 13–48). People also had to choose one of the prescribed languages of daily use on the forms but could refuse to identify with the corresponding ethnolinguistic nation. They could remain non-national and identify with their village or town, valley or province, or religion. For example, Joshua Shanes has shown that many Jews in Galicia never identified with any of the “approved” ethnolinguistic nations even though they could not choose Yiddish or Hebrew as their language of daily use in the census (2012, 36). Last, but not least, people could choose how they wanted to be classified and change their decision as many times as they liked. Their choices were limited by the classification scheme and influenced by nationalists’ propaganda, but in the end they were still theirs.

Nationalists were aware of that and were doing their best to influence people’s identifications. They intensified their efforts; among other things they pressured those they deemed non-national or not national enough (King 2002, 128–129; Zahra 2008, 30–31). The main goal of the nationalists was to remove or at least severely limit people’s agency; they wanted to make nation-ness compulsory and they wanted it ascribed because they basically understood it in essentialist and biological terms. The state had a role to play in these efforts, too. After 1867, the Dualist Hungary was undoubtedly – if not always very successfully – functioning as a nationalizing state that tried to make all its inhabitants into Magyar-speaking Hungarians (Nemes 2016, 8, 9). In contrast, the other half of the Habsburg Monarchy, conventionally called Austria, tried its best to remain non-national. However, such a position became increasingly untenable because nationalists were working hard to involve the state in their plans to give nationhood a quasi-legal status (King 2002, 114, 115). In the first half of the 1870s, nationality – Gerald Stourzh has called it “ethnic attribution” – did enter legal statutes for the first time (Stourzh 2007, 160). Not surprisingly, that opened the doors to legal challenges, with state courts forced to rule on the nationality of individuals. For a while they rejected nationalist assumptions and refused to entertain the idea that nationality could be ascertained objectively. They upheld the so-called *Bekenntnisprinzip*, the principle that nationality can be determined only on the grounds of personal declaration (Stourzh 2007, 161–163).

Later, however, and parallel to the split of many institutions and administrative bodies along national lines, the courts and the state administration started to decide people’s nationality based on supposedly objective markers (Stourzh 2007, 167–172). This was especially important in those provinces where the so-called provincial compromises were reached after the turn of the twentieth century. In Moravia, Bukovina, Galicia, and the Bohemian town of Budweis/Budějovice, national categorization became a requirement, enforced by the bureaucracy and the courts in certain cases. The nationality of individuals was not a matter of individual choice anymore but a matter on which the authorities decided based on supposedly objective criteria (Stourzh 2007, 157–176; Zahra 2008, 13–47). In some instances, the Austrian state was acting as a (multi)nationalizing state.

Because this classification regulated access to schools, voting rights, and some other matters, its impact surpassed that of previous classificatory efforts. It strengthened the boundaries among national groups and made national categories of identification much more relevant. Nevertheless, nation-ness remained a contingent phenomenon and people still had agency even in the provinces where national categorization became obligatory. Classification was still largely their choice, even if it could be legally challenged and could not always be changed; but the room to maneuver was almost always there (Zahra 2008, 13–48). Individual identification with an ethnolinguistic nation remained situational and contingent but enforced classification surely promoted and shaped the spread of nationhood.

Internally displaced people during World War I were a good example. Because the authorities grouped the refugees who were settled in the camps into national categories and their care was largely taken over by national philanthropic organizations, national identification became relevant where it previously hardly mattered. National classification had an impact on the level of care and could – in some instances – even determine if a person would be forced to leave his home and become a refugee or not (Mentzel 1995; Kuprian 2011; Kosi 2016). During the war, national categorization was increasingly important for other civilians, too. Namely, the state put the distribution of food and other aid largely into the hands of private organizations, which were mostly controlled by nationalists (Judson 2016, 426–428).

## Conclusion

In his 2007 article “Nationalist Politics and the Dynamics of State and Civil Society in the Habsburg Monarchy, 1867–1914,” Gary B. Cohen argued,

Nationalist campaigning for group cultural and political rights, including education and other public services in native languages, and government concessions to those demands led gradually to dividing much of public life in the monarchy on linguistic lines and eventually politically articulated national lines. (2007, 261)

This is an excellent portrayal of events; nevertheless, it seems evident that these divisions were not just a result of the nationalists’ pressure and the subsequent concessions of the Habsburg state. They were also driven by state policies that bureaucrats introduced on their own. Their role in establishing national categories of identification, as well as in the recurrent invocation that made them relevant, was more important than previously assumed. This should not come as a surprise if we remember that nation-states, which acted as nationalizing states on purpose, needed a lot of time and effort to make national identification categories relevant for most inhabitants. The notion that national movements could achieve the same effect on their own – or even against – the state, with newspapers, electoral campaigns, gymnastic societies, etc. seems highly unrealistic.

However, we are not advocating for a monocausal and simplistic explanation. Rogers Brubaker has already argued persuasively that we should not conflate a system of classification with the existence of real groups, because institutionalization of national categories does not tell us anything about their resonance. However, he also stressed that “[a] strongly institutionalized ethnonational classificatory system makes certain categories readily and legitimately available for the representation of social reality, the framing of political claims, and the organization of political action. This is itself a fact of great significance” (Brubaker 2006, 53, 54). Brubaker based his claims mostly on developments in the Soviet Union and in the post-Soviet space, and that is a paradigmatic case indeed (Suny and Martin 2001; Hirsch 2005). Post-World War II Yugoslavia is also a good example, as Siniša Malešević has indicated (2006, 158–163). We argue that the formation of nations in the Habsburg Monarchy can be explained in a similar way. The state did not create nations but its classification of languages made available some ethnolinguistic identification categories, which nationalists used to make claims. The institutionalization of these categories also made them more relevant, especially as the nationalist movements simultaneously worked toward the same goal. Furthermore, classification promoted the formation of institutionalized groups. Those groups emphasized differences and helped construct intergroup boundaries.

Yet nation-building did not follow an algorithmic logic. The nations in the post-Habsburg territory correspond to the 1849 linguistic classification to a large degree. Nationalists claim that it simply could not be otherwise because these nations existed long before the onset of nationalism in some way or another; the classification simply reflected reality. However, as historians we know (or at least should know) that it did not, and that the current situation is just one of many possible outcomes. The current situation certainly suggests that the Habsburg linguistic classification from 1849 and its consequent institutionalization had a noticeable influence on the outcome of nation-building. Nevertheless, the obvious exceptions show that other contingencies (strong internal borders, religious differences, etc.) influenced the process as well. That is why we have Czech and Slovaks today and not Czechoslovaks, and have Serbs and Croats and not Illyrians. Still, classification played a role here, too. The military classified Czechs and Slovaks into separate categories. Slovaks were also a separate category in the Hungarian census as were Croats and Serbs. Such an inconsistent classification must have had an impact on the formation of categories of identification. Also, it must be noted, that both exceptions happened in Hungary, where nationalization of the masses was a slow process, not least because of the populations’ severely limited voting rights. Alexander Maxwell has shown that Slovaks are a result of developments that took place after 1918, in the new Czechoslovakia (2009, 166–186).

Finally, it should be noted that the Habsburg bureaucratic classification promoted nationalization and shaped its outcome but nationhood remained contingent and situational. In the beginning of the twentieth century, various nationalisms could mobilize large numbers of people in Austria–Hungary. Yet people still had agency and in some situations they did not act as members of a nation.

## Acknowledgements

This paper is based on the paper we presented at the 2015 World Convention of the Association for the Study of Nationalities. We therefore want to thank our commentator, Vejas Gabriel Liulevicius, and all the participants for their useful comments. We would also like to thank Ágoston Berecz, Tomasz Kamusella, Jernej Kosi, and the anonymous reviewer for their help and advice.

## Notes

1. For the sake of convenience, we will be talking about nations, their formation and existence. However, we agree with Rogers Brubaker that groupism is a pitfall best avoided (2006, 7–27). When we are talking about the existence of nations, we are therefore talking about the existence of an identification category that had significant relevance.

2. For an overview of recent historiography, see Judson (2005) and Cole (2011). For a powerful critique of older historiography, see King (2001).

3. A necessarily arbitrary short selection of case-studies is Verdery (1983), Himka (1988), Moritsch (1991), Cole (2000), Stauter-Halsted (2001), King (2002), Struve (2005), Cohen (2006), Judson (2006), Wingfield (2007), Zahra (2008), Shanes (2012), Almasy (2014a), and Nemes (2016).

4. For a definition of the term, see Brubaker (1996), chap. “Nationalizing State,” 63–66.

5. For a discussion of Hroch’s schema and especially its usefulness in analyzing concurrent and competing nationalisms, see Maxwell (2010).

6. Theodor Domej (2006, 155, 156) shows that the influence of the state had prompted attempts to unify different provincial standards by the last decades of the eighteenth century.

7. The best overview of the language in Austrian censuses is still Brix (1982).
